# Extranodal Male Genital Involvement With Diffuse Large B‐Cell Lymphoma

**DOI:** 10.1155/crh/5511675

**Published:** 2026-05-19

**Authors:** James J. Fradin, Kavanya Feustel, Mehdi Hamadani, Thomas A. Giever

**Affiliations:** ^1^ Division of Internal Medicine, Medical College of Wisconsin, Milwaukee, Wisconsin, USA, mcw.edu; ^2^ Division of Hematology and Oncology, Medical College of Wisconsin, Milwaukee, Wisconsin, USA, mcw.edu

## Abstract

Primary penile diffuse large B‐cell lymphoma (DLBCL) is an exceptionally rare extranodal presentation that can be challenging to diagnose due to its nonspecific symptoms. We report the case of an 84‐year‐old male who initially presented with phimosis who subsequently developed a nonhealing surgical wound and B symptoms. Imaging and biopsy ultimately revealed Stage IVB activated B‐cell type DLBCL. He was treated with dose‐reduced rituximab, cyclophosphamide, doxorubicin, prednisone, and polatuzumab vedotin (modified R‐mini‐CHOP). Despite his advanced disease, he achieved a partial metabolic response with negative minimal residual disease. We also summarize prior reported cases of penile DLBCL, highlighting patterns in disease presentation and treatment approaches. This case emphasizes the importance of maintaining a broad differential for penile lesions to avoid delays in diagnosis and treatment, and it illustrates the integration of novel agents such as polatuzumab vedotin for frail or elderly patients.

## 1. Introduction

Extranodal lymphoma is a well‐documented entity, though certain primary sites are rarer than others [1–3]. Primary genitourinary, and more specifically, primary penile lymphoma is particularly rare, with literature on the topic limited to case reports [4, 5]. Due to the infrequency and often nonspecific initial symptoms, primary genitourinary lymphomas can represent a diagnostic challenge leading to delays in disease‐specific care and poor clinical outcomes. Diffuse large B‐cell lymphoma (DLBCL) is the most common histological subtype of penile lymphoma and can have overall good responses to systemic chemotherapy [6–8]. Here, we present a case of extranodal DLBCL with genital involvement that presented in an atypical fashion, leading to delays in diagnosis and treatment.

## 2. Case Presentation

An 84‐year‐old male with a past medical history of hypertension presented to his urologist in Guam with a chief complaint of urinary retention. Medications included amlodipine, atenolol, losartan, and spironolactone. He had no allergies and was a never‐smoker and never‐drinker. The only family history of malignancy was his son with prostate cancer. He had no known history suggestive of immunodeficiency, immune dysregulation, nor age‐related immunosenescence.

On physical exam, his urologist noted a penile mass and associated swelling. He was diagnosed with phimosis and underwent a partial circumcision. Despite this procedure, he developed progressive growth of the mass and increased swelling over the next 2 months. Computed tomography (CT) of the abdomen and pelvis with contrast revealed multiple masses within the penile shaft and perineum as well as pelvic and inguinal lymphadenopathy.

**FIGURE 1:**
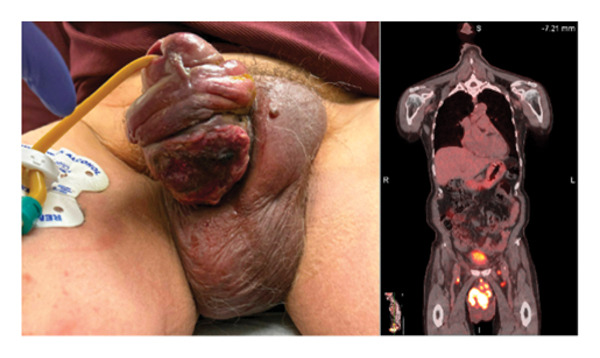
Large ulcerative lesion of the shaft of the penis prior to therapy and positron emission tomography prior to therapy. Note the large hypermetabolic infiltrative mass involving the entirety of the penis.

An indwelling urinary catheter was placed, and he traveled to the United States to seek further care closer to his family. He met with a urologist who noted a large ulcerating proliferative mass on the ventral aspect of the penile shaft with induration and scrotal firmness (Figure 1). Additional perineal masses were noted along with bilateral inguinal lymphadenopathy. He underwent a biopsy of the penile mass and fluorodeoxyglucose‐positron emission tomography (FDG‐PET) imaging (Figure 1) and was referred to medical oncology; given the clinical findings, his presumed diagnosis was advanced penile squamous cell carcinoma (SCC).

FDG‐PET revealed a large hypermetabolic infiltrative mass involving nearly the entirety of the penis (SUV 22.6) which extended into the pelvis with abutment of the prostate, rectum, and urinary bladder. There was hypermetabolic lymphadenopathy above and below the diaphragm with scattered hypermetabolic soft tissue nodules throughout his body. MRI of the brain showed no evidence of CNS metastases.

The biopsy revealed an ulcerated epidermis overlying a dense deep dermal infiltrate composed of sheets of atypical large lymphocytes. Numerous necrotic cells and some mitotic figures were noted. The large, atypical cells were diffusely positive for CD45, CD20, Bcl‐2, BCL6, and MUM‐1. The atypical cells were negative for SOX10, cytokeratin AE1/AE3, CD4, CD123, CD3, CD10, CD30, CD34, and cyclin D1. EBER in situ hybridization was also negative. These findings were most consistent by histology and immunochemistry with large B‐cell lymphoma. Cytogenetics revealed gain of *MYC* in 27% of cells. These findings ultimately led to a diagnosis of Stage IVB DLBCL, activated B‐cell type.

On his initial admission for expedited treatment, vital signs were significant for a temperature of 99°F, heart rate of 73 beats per minute, and blood pressure of 134/68 mmHg. His physical exam was notable for ulcerative lesions at the site of prior penile biopsy with serosanguinous drainage. A firm 5 cm mass was appreciated on the ventral penis with diffuse penile and scrotal edema. There was no notable exudate or purulence from the mass. Laboratory values obtained on admission are noted in Table 1.

**TABLE 1:** Admission laboratory values.

Laboratory	Value	Reference range
*Chemistry*		
Sodium (mmol/L)	130	136–145
Potassium (mmol/L)	4.9	3.4–5.1
Chloride (mmol/L)	91	98–107
Bicarbonate (mmol/L)	28	22–29
Blood Urea Nitrogen (mg/dL)	30	6–23
Creatinine (mg/dL)	1.31	0.70–1.30
Calcium (mg/dL)	11.5	8.6–10.2
Anion Gap (mmol/L)	11	7–15
Glucose (mg/dL)	234	70–180
EGFR (mL/min/1.73 m^2^)	54	≥ 60
Albumin (g/dL)	3.6	3.8–5.0
Total Protein (g/dL)	6.8	6.1–8.2
Alkaline Phosphatase (U/L)	130	40–129
Aspartate Transaminase (U/L)	110	< 50
Alanine Transaminase (U/L)	17	< 42
Total Bilirubin (mg/dL)	1.2	0.2–1.2
LDH (U/L)	1919	135–225

*Complete Blood Count*		
White Blood Cell (10^3^/μL)	14.6	4.0–10.0
Red Blood Cell (10^6^/μL)	4.1	4.4–5.9
Hemoglobin (g/dL)	12.8	13.7–17.5
Hematocrit (%)	38	40–51
Mean Corpuscular Volume (fL)	92	79–98
Platelet Count (10^3^/μL)	202	160–400

*Differential*		
Neutrophil (%)	85	43–74
Lymphocyte (%)	5	17–46
Monocyte (%)	9	4–13
Eosinophil (%)	0	0–6
Basophil (%)	0	0‐1
Immature Granulocyte (%)	1	0‐1

*Coagulation*		
Prothrombin Time (s)	10.3	9.5–11.8
International Normalized Ratio	1.0	N/A

*Note:* Significant findings include elevated LDH, leukocytosis, and hypercalcemia.

He started standard infection prophylaxis with acyclovir and trimethoprim‐sulfamethoxazole, as well as therapeutic amoxicillin‐clavulanate for a urinary tract infection. Allopurinol was initiated for tumor lysis syndrome prophylaxis. Due to his age, systemic chemotherapy with a reduced‐dose regimen of rituximab, cyclophosphamide, doxorubicin, vincristine, and prednisone (R‐mini‐CHOP) was recommended.

He had no complications following initiation of chemotherapy. His second cycle of R‐mini‐CHOP was modified to include polatuzumab vedotin in place of vincristine. Following 6 cycles of R‐mini‐CHP plus polatuzumab vedotin, he attained a partial metabolic response (Deauville 4) by PET‐CT and was negative for minimal residual disease by clonoSEQ (measurable residual disease testing). He has since received an additional 2 post‐treatment PET‐CT scans showing stable FDG‐avid mediastinal lymphadenopathy which is favored to be reactive which would improve his response to a complete response (Deauville 1) at 8 months from the end of treatment.

## 3. Discussion

Penile cancer is exceedingly rare, representing < 1% of all cancer diagnoses in the United States and Europe [9]. The most common penile cancer is SCC, which can present with similar signs and symptoms similar to penile lymphoma. The management of penile SCC is evolving but traditionally utilizes a combination of chemotherapy and surgical resection, which can lead to significant disfigurement and psychosocial distress in addition to potential functional complications [9]. Penile lymphoma was first reported in 1962 [10], and, more specifically, the first case of penile DLBCL was reported in 1988 [11]. The most common histological subtype of penile lymphoma is DLBCL [6, 7].

The outcomes for penile DLBCL can be quite favorable given most cases are reported in early stages. In a review of 20 cases, 63.2% of patients were Stage IE at diagnosis and Stage IV was not reported, indicating most DLBCL originating from the penis are diagnosed and treated in the localized setting [6]. Initial treatment responses are good, with a complete response rate of 90% and 12 month progression‐free survival of 76.5% [6]. Compared with nodal DLBCL in older age, primary DLBCL of the penis was more sensitive to chemotherapy and associated with a better progression‐free survival rate [6]. In a review of SEER database data, patients with primary genitourinary lymphoma were found to have a median survival of 76 months [3].

There is no consensus standard treatment for penile lymphoma. First‐line systemic chemotherapy is most often R‐CHOP or other related regimens [7]. In the treatment of DLBCL, R‐CHOP is successful in treating lymphoma in approximately 60% of patients [8]. In patients of an advanced age or impaired functional status, dose‐attenuated versions of R‐CHOP therapies can be reasonable alternatives (i.e., R‐mini‐CHOP) [12, 13]. Radiotherapy may be used adjunctively, and surgical intervention is considered typically only for refractory or recurrent cases, with organ‐sparing approaches preferred when feasible due to the psychosocial and functional implications of excision of penile masses and penectomies [14].

Of the 27 published, MEDLINE‐indexed, cases of extranodal DLBCL with penile involvement, the median age at diagnosis was 65 years old, and the most common stage at diagnosis was IE (*n* = 12) (Supporting Table 1). The most common presenting symptom was a penile mass with or without ulceration (*n* = 11). Other less common presenting symptoms included dysuria (*n* = 3), hematuria (*n* = 2), priapism (*n* = 3), and edema (*n* = 1). The most common treatment regimen used was cyclophosphamide, doxorubicin, vincristine, and prednisone (CHOP) with (*n* = 12) or without (*n* = 9) rituximab.

In our case, clonoSEQ (a circulating tumor DNA assay) was used as an additional marker of treatment response. Typically, response is measured by CT or FDG‐PET, but circulating tumor DNA has gained popularity due to limitations of imaging to fully assess treatment response [15, 16]. Multiple recent prospective studies have proven that these assays can independently predict improved survival and outperform PET/CT imaging in detecting subclinical levels of disease [17–19]. In our case, this minimal residual disease testing was useful in assessing treatment response in the presence of residual FDG avid mediastinal lymphadenopathy and the patient’s preference to defer biopsy.

Our case report of penile extranodal DLBCL has several unique features. The patient had a very significant delay in his final diagnosis, most likely leading to more advanced disease prior to treatment. He relocated from Guam to the United States, adding an additional delay in evaluation and management as he navigated the change in health systems. His presentation was also atypical. Phimosis is an uncommon presenting sign of penile DLBCL. Some authors have proposed a broader association between phimosis and penile SCC involving retention of epidermal cells and urinary products leading to chronic irritation and inflammation, predisposing patients to malignancy [20]. Studies have shown 44%–85% of patients with penile SCC also had phimosis and that the relative risk of penile cancer was increased in males with phimosis [21, 22]. DLBCL can also arise in the setting of inflammation. DLBCL associated with chronic inflammation (DLBCL‐CI) is its own subtype and has been associated with EBV‐positive lymphoma and pyothorax [23, 24], but our patient had no known prior chronic inflammatory state. Although he presented with Stage IV disease, a reduced intensity chemotherapy regimen was selected (R‐mini‐CHOP) due to his modest functional status and advanced age. Polatuzumab vedotin was exchanged for vincristine starting with the second cycle due to evidence from the POLARIX trial which showed decreased disease progression, relapse, and death as compared to patients receiving traditional R‐CHOP [25]. Point estimates from this trial suggested improved response to Pola‐R‐CHP in patients > 60 years old and with activated B‐cell subtype lymphoma, similar to the characteristics of our patient [25]. A recent retrospective cohort study of 172 patients with DLBCL treated with frontline Pola‐R‐CHP in Japanese hospitals found that objective response rate (ORR) was similar in patients ≥ 80 years old (ORR 89.5%) and < 80 years old (ORR 97.3%), but with increased treatment‐related mortality in the older age group (11.4% vs. 2.3%) [26]. These findings underscore the potential role of polatuzumab vedotin in the treatment of elderly patients with DLBCL.

In summary, this case highlights the importance of considering lymphoma in the differential diagnosis of atypical penile lesions. Early biopsy and broad diagnostic consideration are critical to avoid delays in treatment. Standard reduced‐intensity regimens for DLBCL may offer clinical benefit for elderly patients with advanced‐stage disease, and polatuzumab vedotin may be an attractive option in future treatment of extranodal lymphoma.

## Funding

This work received no external funding.

## Disclosure

All authors have read and approved the final version of the manuscript. James J. Fradin had full access to all of the data in this study and takes complete responsibility for the integrity of the data and the accuracy of the data analysis.

## Consent

No patient identifiers were used, and the patient was sufficiently anonymized according to ICMJE guidelines. The patient provided informed written consent prior to publication.

## Conflicts of Interest

The authors declare no conflicts of interest.

## Supporting Information

Additional supporting information can be found online in the Supporting Information section.

## Supporting information


**Supporting Information** File 1: Table of prior cases of DLBCL with penile involvement.

## Data Availability

The data that support the findings of this study are available from the corresponding author upon reasonable request.

## References

[1] Arambulo S. , Calle A. , Vela J. M. , and Sotelo M. J. , Advanced Penile Lymphoma: Case Report and Review of the Literature, Journal of Cancer Research and Therapeutics. (2023) 19, no. 3, 823–825, 10.4103/jcrt.jcrt_593_21. 37470619

[2] Krol A. D. G. , le Cessie S. , Snijder S. , Kluin-Nelemans J. C. , Kluin P. M. , and Noordijk E. M. , Primary Extranodal Non-Hodgkin’s Lymphoma (NHL): The Impact of Alternative Definitions Tested in the Comprehensive Cancer Centre West Population-Based NHL Registry, Annals of Oncology. (2003) 14, no. 1, 131–139, 10.1093/annonc/mdg004, 2-s2.0-12244305835. 12488305

[3] Gupta V. , Singh V. , Bajwa R. et al., Site-Specific Survival of Extra Nodal Diffuse Large B-Cell Lymphoma and Comparison With Gastrointestinal Diffuse Large B-Cell Lymphoma, International Journal of Hematology. (2022) 11, no. 2, 45–54, 10.14740/jh984. PMC9076143 35573751

[4] Vallatharasu Y. , Chennamadhavuni A. , and Van Every M. J. , Twenty-Year Experience With Genitourinary Lymphoma at a Community Hospital, Clinical Medicine and Research. (2021) 19, no. 2, 72–81, 10.3121/cmr.2021.1531. 33789953 PMC8231695

[5] Schniederjan S. D. and Osunkoya A. O. , Lymphoid Neoplasms of the Urinary Tract and Male Genital Organs: A Clinicopathological Study of 40 Cases, Modern Pathology. (2009) 22, no. 8, 1057–1065, 10.1038/modpathol.2009.65, 2-s2.0-68249119682. 19377442

[6] Yu T. , Zou L. , Wang Y. , Luo C. , and Yu L. , Primary Diffuse Large B-Cell Lymphoma of the Penis: A Case and Literature Review, OncoTargets and Therapy. (2023) 16, 631–638, 10.2147/OTT.S408195. 37520144 PMC10386859

[7] Chu L. , Mao W. , Curran Vikramsingh K. et al., Primary Malignant Lymphoma of the Glans Penis: A Rare Case Report and Review of the Literature, Asian Journal of Andrology. (2013) 15, no. 4, 571–572, 10.1038/aja.2013.21, 2-s2.0-84880111549. 23644872 PMC3739230

[8] Sehn L. H. and Salles G. , Diffuse Large B-Cell Lymphoma, New England Journal of Medicine. (2021) 384, no. 9, 842–858, 10.1056/NEJMra2027612. 33657296 PMC8377611

[9] Douglawi A. and Masterson T. A. , Updates on the Epidemiology and Risk Factors for Penile Cancer, Translational Andrology and Urology. (2017) 6, no. 5, 785–790, 10.21037/tau.2017.05.19, 2-s2.0-85032280994. 29184774 PMC5673812

[10] Oomura J. , Ookita K. , Takenaka M. , and Yamada S. , [Primary Reticulosarcoma of the Penis. Report of a Case], Hinyokika Kiyo. (1962) 8, 536–542. 13940193

[11] Marks D. , Crosthwaite A. , Varigos G. , Ellis D. , and Morstyn G. , Therapy of Primary Diffuse Large Cell Lymphoma of the Penis With Preservation of Function, The Journal of Urology. (1988) 139, no. 5, 1057–1058, 10.1016/s0022-5347(17)42771-6, 2-s2.0-0023884546. 3361644

[12] Peyrade F. , Jardin F. , Thieblemont C. et al., Attenuated Immunochemotherapy Regimen (R-MiniCHOP) in Elderly Patients Older Than 80 Years With Diffuse Large B-Cell Lymphoma: A Multicentre, Single-Arm, Phase 2 Trial, Lancet Oncology. (2011) 12, no. 5, 460–468, 10.1016/S1470-2045(11)70069-9, 2-s2.0-79955473473. 21482186

[13] Al-Sarayfi D. , Brink M. , Chamuleau M. E. D. et al., R-MiniCHOP Versus R-CHOP in Elderly Patients With Diffuse Large B-Cell Lymphoma: A Propensity Matched Population-Based Study, American Journal of Hematology. (2024) 99, no. 2, 216–222, 10.1002/ajh.27151. 38014799

[14] el-Sharkawi A. and Murphy J. , Primary Penile Lymphoma: The Case for Combined Modality Therapy, Clinical Oncology. (1996) 8, no. 5, 334–335, 10.1016/s0936-6555(05)80726-5, 2-s2.0-0029802196. 8934056

[15] Adams H. J. A. , Nievelstein R. A. J. , and Kwee T. C. , Prognostic Value of Complete Remission Status at End-of-Treatment FDG-PET in R-CHOP-Treated Diffuse Large B-Cell Lymphoma: Systematic Review and Meta-Analysis, British Journal of Haematology. (2015) 170, no. 2, 185–191, 10.1111/bjh.13420, 2-s2.0-84934436398. 25833790

[16] Cheson B. D. , Fisher R. I. , Barrington S. F. et al., Recommendations for Initial Evaluation, Staging, and Response Assessment of Hodgkin and Non-Hodgkin Lymphoma: The Lugano Classification, Journal of Clinical Oncology. (2014) 32, no. 27, 3059–3068, 10.1200/JCO.2013.54.8800, 2-s2.0-84903462466. 25113753 PMC4979083

[17] Wang S. , Nijland M. , Strobbe L. et al., Prospective Validation of Circulating Tumor DNA Measurable Residual Disease After First-Line Therapy in Large B-Cell Lymphoma, Journal of Clinical Oncology. (2026) 44, no. 5, 400–409, 10.1200/JCO-25-01712. 41385760 PMC12879184

[18] Roschewski M. , Kurtz D. M. , Westin J. R. et al., Remission Assessment by Circulating Tumor DNA in Large B-Cell Lymphoma, Journal of Clinical Oncology. (2025) 43, no. 34, 3652–3661, 10.1200/JCO-25-01534. 40802906 PMC12363663

[19] Krupka J. A. , Moutsopoulos I. , Cutmore N. H. et al., Phased Variant-Supported Circulating Tumor DNA as a Prognostic Biomarker After First-Line Treatment in Large B-Cell Lymphoma: Findings From the DIRECT Study, Journal of Clinical Oncology. (2026) 44, no. 5, 410–420, 10.1200/JCO-25-01587. 41428995

[20] Bleeker M. C. G. , Heideman D. A. M. , Snijders P. J. F. , Horenblas S. , Dillner J. , and Meijer C. J. L. M. , Penile Cancer: Epidemiology, Pathogenesis and Prevention, World Journal of Urology. (2009) 27, no. 2, 141–150, 10.1007/s00345-008-0302-z, 2-s2.0-63649084378. 18607597

[21] Daling J. R. , Madeleine M. M. , Johnson L. G. et al., Penile Cancer: Importance of Circumcision, Human Papillomavirus and Smoking in in Situ and Invasive Disease, International Journal of Cancer. (2005) 116, no. 4, 606–616, 10.1002/ijc.21009, 2-s2.0-23244462578. 15825185

[22] Dillner J. , von Krogh G. , Horenblas S. , and Meijer C. J. , Etiology of Squamous Cell Carcinoma of the Penis, Scandinavian Journal of Urology and Nephrology—Supplementum. (2000) 205, 189–193, 10.1080/00365590050509913. 11144896

[23] Sugita Y. , Masuoka J. , Kameda K. et al., Primary Central Nervous System Lymphomas Associated With Chronic Inflammation: Diagnostic Pitfalls of Central Nervous System Lymphomas, Brain Tumor Pathology. (2020) 37, no. 4, 127–135, 10.1007/s10014-020-00373-z. 32627089

[24] Loong F. , Chan A. C. L. , Ho B. C. S. et al., Diffuse Large B-Cell Lymphoma Associated With Chronic Inflammation as an Incidental Finding and New Clinical Scenarios, Modern Pathology. (2010) 23, no. 4, 493–501, 10.1038/modpathol.2009.168, 2-s2.0-77950517464. 20062008

[25] Tilly H. , Morschhauser F. , Sehn L. H. et al., Polatuzumab Vedotin in Previously Untreated Diffuse Large B-Cell Lymphoma, New England Journal of Medicine. (2022) 386, no. 4, 351–363, 10.1056/NEJMoa2115304. 34904799 PMC11702892

[26] Yamada T. , Nakamura N. , Goto H. et al., Polatuzumab Vedotin, Rituximab, Cyclophosphamide, Doxorubicin, and Prednisone (Pola-R-CHP) Therapy in Diffuse Large B-Cell Lymphoma in Patients Aged 80 Years or Older: A Real-World Study, Annals of Hematology. (2025) 104, no. 10, 5191–5200, 10.1007/s00277-025-06619-0. 41068304 PMC12619725

