# Supplementary material for: Extranodal Male Genital Involvement With Diffuse Large B‐Cell Lymphoma
Source: Case Rep Hematol. 2026 May 19;2026:5511675. doi: 10.1155/crh/5511675 (PMC13189451; doi:10.1155/crh/5511675)
Supplement: Supplementary file 1 — Supporting Information File 1: Table of prior cases of DLBCL with penile involvement. [file CRH-2026-5511675-s001.docx]

**Supplemental Figures**

| **Case** | **Age** | **Clinical Presentation** | **Stage** | **MYC Status** | **Treatment** | **Regimen** | **Response** | **Reference** |
| --- | --- | --- | --- | --- | --- | --- | --- | --- |
| 1 | 65 | Erythematous penile mass | IE | NR | Systemic chemo | CHOP x 2 | Alive and free of disease at time of publication: May 1988 | Marks D, Crosthwaite A, Varigos G, Ellis D, Morstyn G. Therapy of primary diffuse large cell lymphoma of the penis with preservation of function. *J Urol*. **1988**;139(5):1057-1058. doi:10.1016/s0022-5347(17)42771-6 |
| 2 | 66 | Erythematous/ulcerated penile nodule | IE | NR | Radiation | 44Gy in 22 fractions | Alive and free of disease for 12 months at the time of publication (Jun 1994) | Hashine K, Akiyama M, Sumiyoshi Y. Primary diffuse large cell lymphoma of the penis. *Int J Urol*. **1994**;1(2):189-190. doi:10.1111/j.1442-2042.1994.tb00035.x |
| 3 | 18 | Painless penile ulcer | IE | NR | Systemic chemo | CHOP x 8 | Alive and free of disease for 27 months at the time of publication (Mar 1995) | Fairfax CA, Hammer CJ 3rd, Dana BW, Hanifin JM, Barry JM. Primary penile lymphoma presenting as a penile ulcer. *J Urol*. **1995**;153(3 Pt 2):1051-1052. |
| 4 | 82 | Nocturia, urinary hesitancy, penile mass | IE | NR | Surgery; systemic chemo | Penile amputation  Post-Relapse 27.5 Gy in 10 fractions, COP x 6 | Relapsed 12 months following amputation, then patient died “a few months later” | el-Sharkawi A, Murphy J. Primary penile lymphoma: the case for combined modality therapy. *Clin Oncol (R Coll Radiol)*. **1996**;8(5):334-335. doi:10.1016/s0936-6555(05)80726-5 |
| 5 | 64 | Painful penile mass | IIE | NR | Systemic chemo | CHOP x 5 | Death 7 months following treatment due to progressive disease | Kuwahara Y, Kubota Y, Hibi H, et al. *Hinyokika Kiyo*. **1997**;43(5):371-374. |
| 6 | 63 | Painless penile mass | IIIE | NR | Systemic chemo; radiation; IT chemo | CHOP x 4, radiation 30Gy | Alive and free of disease for 10 months at the time of publication (May 1997) | Kuwahara Y, Kubota Y, Hibi H, et al. *Hinyokika Kiyo*. **1997**;43(5):371-374. |
| 7 | 75 | Diffuse penile mass | IE | NR | Systemic chemo; radiation | MINE x 3; 40Gy | Alive and free of disease for 42 months at the time of publication (Feb 2001) | Arena F, di Stefano C, Peracchia G, Barbieri A, Cortellini P. Primary lymphoma of the penis: diagnosis and treatment. *Eur Urol*. **2001**;39(2):232-235. doi:10.1159/000052441 |
| 8 | 64 | Erectile dysfunction | IE | NR | Systemic chemo; radiation | CHOP x 3, 40Gy to base of penis, 36Gy to lymph nodes  Post-Relapse:  MINE x 3, 36Gy to entire penis shaft, rituximab, WBRT and MTX | Relapsed 1 year following CHOP + radiation as new penile mass.  Response following MINE and radiation, but found to have diffuse cerebral involvement  Stable disease at the time of publication (Jun 2001) | Beal K, Mears JG. Short report: penile lymphoma following local injections for erectile dysfunction. *Leuk Lymphoma*. **2001**;42(1-2):247-249. doi:10.3109/10428190109097700 |
| 9 | 45 | Fever, lower abdominal pain, painless penile ulcers | IIIE | NR | Systemic chemo | CHOP x 6 | Alive and free of disease for 6 months at the time of publication (Aug 2005) | Jabr FI. Recurrent lymphoma presenting as a penile ulcer in a patient with AIDS. *Dermatol Online J*. **2005**;11(2):29. Published 2005 Aug 1. |
| 10 | 50 | Priapism | IIE | NR | Systemic chemo | CHOP | NR | Madeb R, Rub R, Erlich N, Hegarty PK, Yachia D. Long standing priapism as presentation of lymphoma. *Am J Hematol*. **2007**;82(1):87. doi:10.1002/ajh.20810 |
| 11 | 77 | Painful penile mass | IVE | NR | Systemic chemo | R-CHOP | Alive and free of disease at the time of publication (Jul 2008) | Vassou A, Bai M, Benetatos L, Tsili A, Bourantas K. Large B-cell transformation of chronic lymphocytic leukemia presenting as a penile mass and skin lesion. *Hematol Oncol Stem Cell Ther*. **2008**;1(3):199-200. doi:10.1016/s1658-3876(08)50032-x |
| 12 | 42 | Hematuria, painless palpable penile mass | IE | NR | Systemic chemo | R-CHOP x 6 | Alive and free of disease for 7 months at time of publication (Nov 2008) | Kim HY, Oh SY, Lee S, et al. Primary penile diffuse large B cell lymphoma treated by local excision followed by rituximab-containing chemotherapy. *Acta* *Haematol*. 2008;120(3):150-152. doi:10.1159/000178146 |
| 13 | 74 | Painless swelling of penis | IE | NR | Systemic chemo | R-CHOP x 4, Rituximab x 4 | Alive and free of disease for 30 months at time of publication (Apr 2009) | Gallardo F, Pujol RM, Barranco C, Salar A. Progressive painless swelling of glans penis: uncommon clinical manifestation of systemic non-Hodgkin's lymphoma. *Urology*. **2009**;73(4):929.e3-929.e929005.doi:10.1016/j.urology.2008.04.059 |
| 14 | 71 | NR | NR | NR | Systemic chemo; radiation | NR | Alive and free of disease for 48 months at time of publication (Jul 2009) | Ibarz Servio L, Arzoz Fábregas M, Ruiz Domínguez JM, Batlle Massana M, Mate Sanz JL, Saladié Roig JM. Linfoma primario de pene [Primary lymphoma of penis]. *Actas Urol Esp*. **2009**;33(7):826-829. doi:10.1016/s0210-4806(09)74238-x |
| 15 | 67 | Dysuria, gross hematuria | IIE | NR | Systemic chemo | R-CHOP x 8 | Alive and free of disease for 10 months at time of publication (Apr 2012) | Hamamoto S, Tozawa K, Nishio H, Kawai N, Kohri K. Successful treatment of primary malignant lymphoma of the penis by organ-preserving rituximab-containing chemotherapy. *Int J Clin Oncol*. **2012**;17(2):181-184. doi:10.1007/s10147-011-0273-8 |
| 16 | 73 | Painless penile mass | IE | NR | Systemic chemo | CHOP x 2 | Alive and free of disease for 33 months at time of publication (Dec 2012) | Wang GC, Peng B, Zheng JH. Primary penile malignant lymphoma: report of a rare case. *Can Urol Assoc J*. **2012**;6(6):E277-E279. doi:10.5489/cuaj.11299 |
| 17 | 76 | Fever, priapism | IVE | NR | Systemic chemo | R-CHOP x 6, IT MTX x 4  Post-Relapse: R-MTX-TMZ x 3 | CNS relapse 6 months following R-CHOP, IT-MTX.  Reported significant clinical improvement after T-MTX-TMZ. | Wakim JJ, Levenson BM, Mathews D, Naina HV. Management of an unusual case of intravascular large B-cell lymphoma of the penis, prostate, and bones with CNS relapse. *J Clin Oncol*. **2013**;31(17):e288-e290. doi:10.1200/JCO.2012.46.6003 |
| 18 | 63 | Painless penile swelling, dysuria, urinary retention | IIE | NR | Systemic chemo | R-CHOP x 8 | Alive and free of disease for 18 months at time of publication (Mar 2013). | Karunanithi S, Sharma P, Naswa N, et al. Primary penile lymphoma: the use of PET-CT for accurate staging and response monitoring. *Diagn Interv Radiol*. **2013**;19(2):130-133. doi:10.4261/1305-3825.DIR.6253-12.1 |
| 19 | 49 | Penile swelling, double stream, post-micturition dribbling, urethral stricture, non-healing penile ulcer | IE | NR | Systemic chemo | CHOP x 6 | Alive and free of disease at time of publication (Jul 2013). | Karki K, Mohsin R, Mubarak M, Hashmi A. Primary Non-Hodgkin's Lymphoma of Penis Masquerading as a Non-Healing Ulcer in the Penile Shaft. *Nephrourol Mon*. **2013**;5(3):840-842. doi:10.5812/numonthly.6885 |
| 20 | 48 | Priapism | IVE | NR | Systemic chemo; radiation | E-CHOP x 1, then radiation | Death 3 months after diagnosis | Gong Z, Zhang Y, Chu H, et al. Priapism as the initial symptom of primary penile lymphoma: A case report. *Oncol Lett*. **2014**;8(5):1929-1932. doi:10.3892/ol.2014.2488 |
| 21 | 51 | Penile swelling, painless penile ulcerations, dysuria, urinary frequency | IIIE | NR | Systemic chemo | R-CHOP | Receiving treatment at time of publication (Dec 2016). | Öneç B, Öneç K, Esbah AÜ, Esbah O. Presentation of Diffuse Large B-Cell Lymphoma Relapse as a Penile Mass. Penil Kitle ile Başvuran Diffüz Büyük B Hücreli Lenfoma Nüksü. *Turk J Haematol*. **2016**;33(4):362-363. doi:10.4274/tjh.2016.0132 |
| 22 | 78 | Painful penile swelling and ulceration | IIE | None | Systemic chemo | R-CHOP x 6, then BR x 6  Post-Relapse, R-Ibr x 6 | Length of first response unknown, relapsed, then alive and free of disease for 4 months at time of publication (May 2016) | Yamany T, Reddy BY, Husain S, Grossman ME. Recurrent Richter's Transformation Presenting With a Penile Ulcer. *JAMA Dermatol*. **2016**;152(5):586-587. doi:10.1001/jamadermatol.2015.5708 |
| 23 | 76 | Penile ulceration, swelling, nodules | IE | None | Systemic chemo; radiation | R-THP-COP x 3  Radiation 40 Gy in 20 fractions  Post-relapse: WBRT | Relapsed 2 years following R-THP-COP with a pituitary stalk lesion.  Alive and free of disease for 3 months at time of publication (Apr 2017). | Tanaka Y, Tanaka A, Hashimoto A, Shinzato I. Isolated Pituitary Stalk Relapse of Primary Penile Lymphoma. *Intern Med*. **2017**;56(7):835-839. doi:10.2169/internalmedicine.56.7788 |
| 24 | 57 | Painless penile mass | IIE | None | Systemic chemo | R-CHOP x 6 | Alive and free of disease at time of publication (Nov 2021). | Fenech M, Pisani D, Camilleri DJ. Primary high-grade diffuse large B-cell lymphoma of the glans penis. *BMJ Case Rep*. **2021**;14(11):e243844. Published 2021 Nov 12. doi:10.1136/bcr-2021-243844 |
| 25 | 59 | Pain with erection | IIE | NR | Systemic chemo | R-CHOP x 8, then Rituximab x 1 | Alive and free of disease for 2 years at time of publication (Nov 2022). | Diao L, Yang S, Shang P, Hou Z. Report of penis lymphoma and review of the literature. *Asian J Surg*. **2022**;45(11):2528-2529. doi:10.1016/j.asjsur.2022.05.136 |
| 26 | 79 | Painful, erythematous, edematous penile lesion | IIE | NR | Systemic chemo | R-CVP, then R-CHOP x 6 | Alive and free of disease for 3 years at time of publication (Nov 2022). | Diao L, Yang S, Shang P, Hou Z. Report of penis lymphoma and review of the literature. *Asian J Surg*. **2022**;45(11):2528-2529. doi:10.1016/j.asjsur.2022.05.136 |
| 27 | 86 | Penile ulcer | IE | None | Systemic chemo; surgical resection | R-COP x 2, then R-mini-CHOP x 1  Post-Relapse: penectomy | Alive and free of disease at time of publication (Jul 2023). | Yu T, Zou L, Wang Y, Luo C, Yu L. Primary Diffuse Large B-Cell Lymphoma of the Penis: A Case and Literature Review. *Onco Targets Ther*. 2023;16:631-638. Published **2023** Jul 25. doi:10.2147/OTT.S408195 |

**Supplemental Table 1. Prior Cases of DLBCL with Penile Involvement Reported in** **the Literature.** Abbreviations: BR – bendamustine, rituximab; chemo – chemotherapy; CHOP – cyclophosphamide, doxorubicin, vincristine, prednisone; COP – cyclophosphamide, vincristine, prednisone; E-CHOP – etoposide, cyclophosphamide, doxorubicin, vincristine, prednisone; IT – intrathecal; MINE – mesna, ifosfamide, mitoxantrone, etoposide; MTX – methotrexate; NR - not reported; R-CHOP – rituximab, cyclophosphamide, doxorubicin, vincristine, prednisone; R-COP – rituximab, cyclophosphamide, vincristine, prednisone; R-CVP – rituximab, cyclophosphamide, vincristine, prednisone; R-Ibr – rituximab, ibrutinib; R-MTX-TMZ – rituximab, methotrexate, temozolomide; R-THP-COP – rituximab, pirarubicin, cyclophosphamide, vincristine, prednisone; WBRT – whole brain radiation therapy.
